# Aripiprazole-induced liver injury: a spontaneous reporting database study

**DOI:** 10.3389/fphar.2023.1226386

**Published:** 2023-08-24

**Authors:** Yunjuan Gao, Chengzhao Wu, Xingran Zhai, Ming Niu, Zhaofang Bai, Haibo Song, Xu Zhao, Jiabo Wang, Xiaohe Xiao

**Affiliations:** ^1^ School of Pharmacy, Chengdu University of Traditional Chinese Medicine, Chengdu, China; ^2^ Fifth Medical Center of Chinese PLA General Hospital, Beijing, China; ^3^ National Center for Adverse Drug Reaction Monitoring, Beijing, China; ^4^ School of Traditional Chinese Medicine, Capital Medical University, Beijing, China

**Keywords:** aripiprazole, antipsychotic drugs, drug-induced liver injury, risk identification, safe medication

## Abstract

**Background:** There have been individual case reports of aripiprazole in recent years, both domestically and internationally, but no analysis of the characteristics of the occurrence of adverse reactions/events of drug-induced liver injury with aripiprazole using spontaneous reports has been seen.

**Methods:** Using a retrospective study approach, the 452 adverse reaction/event reports of aripiprazole-induced liver injury collected by the China Adverse Drug Reaction Monitoring System from 1 January 2012 to 31 December 2016 were analyzed and evaluated, and exploring it’s the clinical characteristics and related risk factors for liver injury occurrence.

**Results:** Among 452 cases of aripiprazole-induced liver injury ADR/ADE reports, there were 121 cases classified as serious, accounting for 26.8% of the total. There were 250 male and 202 female patients, with a male-to-female ratio of 1.24:1. The age of patients ranged from 11 to 77 years old, with an average age of (34.56 ± 12.81) years old, and a high proportion of young adults in the total population. Some patients had used the drug off-label or at a higher than recommended dosage. The onset of liver injury was generally within 15–90 days after continuous use, while some patients are also accompanied by nausea, vomiting, and weight gain. 70% of the combined drug instructions listed that may cause liver injury.

**Conclusion:** In clinical practice, healthcare professionals should pay closely attention to the adverse reactions and risk factors of liver injury caused by aripiprazole. If there are potential risk factors for liver injury, early and regular monitoring of liver function should be carried out to reduce the occurrence of adverse reactions.

## Introduction

Aripiprazole is a novel atypical antipsychotic drug with unique pharmacological characteristics and is used extensively in clinical practice. It is used in the treatment of schizophrenia and bipolar disorder type-I mania because of its partial activation of the D2 dopamine receptor and 5-hydroxytryptamine (serotonin) 1A receptor and its antagonistic effect on 5-HT_2A_ (serotonin) receptor. Its side effects are mild. The US website LiverTox notes that aripiprazole’s hepatotoxicity likelihood score falls under the E category, namely, unlikely cause of clinically apparent liver injury (liverTox, 2017). However, a literature search reveals that several cases related to aripiprazole-induced liver injury have been reported so far both in China and internationally (Castanheira et al., 2019; Pirc et al., 2020; Wang et al., 2018; Tang et al., 2005; Nikogosyan et al., 2021), and there are no large-scale epidemiological studies that are available to provide sufficient pharmacoepidemiology evidence. A basis for estimating aripiprazole-associated hepatotoxicity adverse drug reaction (ADR) risk and measures for its clinical prevention and control are still lacking. Therefore, this study retrospectively analyzed the aripiprazole-induced liver injury ADR reports based on the China National Adverse Drug Reactions Monitoring System, in order to provide reference for safe medication in clinical practice.

## Materials and methods

### Data sources and extraction screening

**FIGURE 1:**
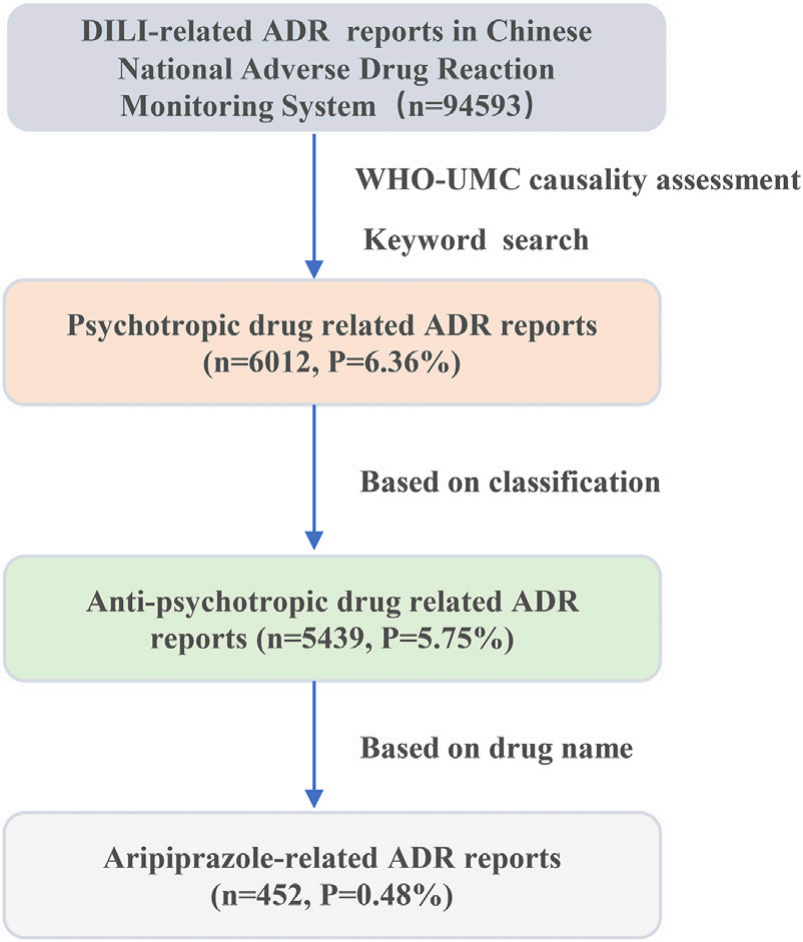
A flowchart for aripiprazole-induced liver injury ADR reports analysis.

The original dataset was derived from our previous research, which consisted of the collected details from suspected 94,593 ADR records reported to liver injury in the Chinese National Adverse Drug Reaction Monitoring System from 1 January 2012, to 31 December 2016. The included reports were categorized as liver injury-related ADR, such as “drug-induced liver injury,” “drug-induced liver damage,” and “abnormal liver function caused by drug”. Meanwhile, reports with keywords indicating non-drug etiology, such as “viral liver disease,” “alcoholic liver disease,” and “autoimmune liver disease” were excluded. Additionally, incomplete records and reports with the WHO Uppsala Monitoring Centre (UMC) causality judgement of “possibly not” or “not” were excluded as well (Uppsala Monitoring Centre, 1997; Wang et al., 2022). Based on this dataset, we futher performed a step-by-step search using the keywords “psychotropic” and “antidepressant medications” to identify, and finally screen out the reports related to aripiprazole for analysis, as shown in Figure 1.

### Outline of the study

We conducted a retrospective study to analyze 452 ADR reports of liver injury induced by aripiprazole from the aspects of patient information, drug information, and occurrence of adverse reactions. The specific variables we considered included the patient’s gender, age, underlying diseases, reasons for medication, dosage and drug combinations, time of occurrence of adverse reactions, recovery, and prognosis. Additionally, we analyzed the clinical characteristics and related risk factors of adverse reactions in the context of a review of relevant literature in China and abroad.

### Statistical analysis

The data were extracted, collated, cleaned, and summarized using Excel 2019, and we used SPSS 26.0 for the statistical analysis. We adopted descriptive epidemiological methods; continuous variables are statistically described using mean ± standard deviation ( $\bar{x}\pm s$ ); numerical count data cases are expressed as percentage (%).

## Results

### Basic information of ADR reports of aripiprazole-induced liver injury

#### General details

**FIGURE 2:**
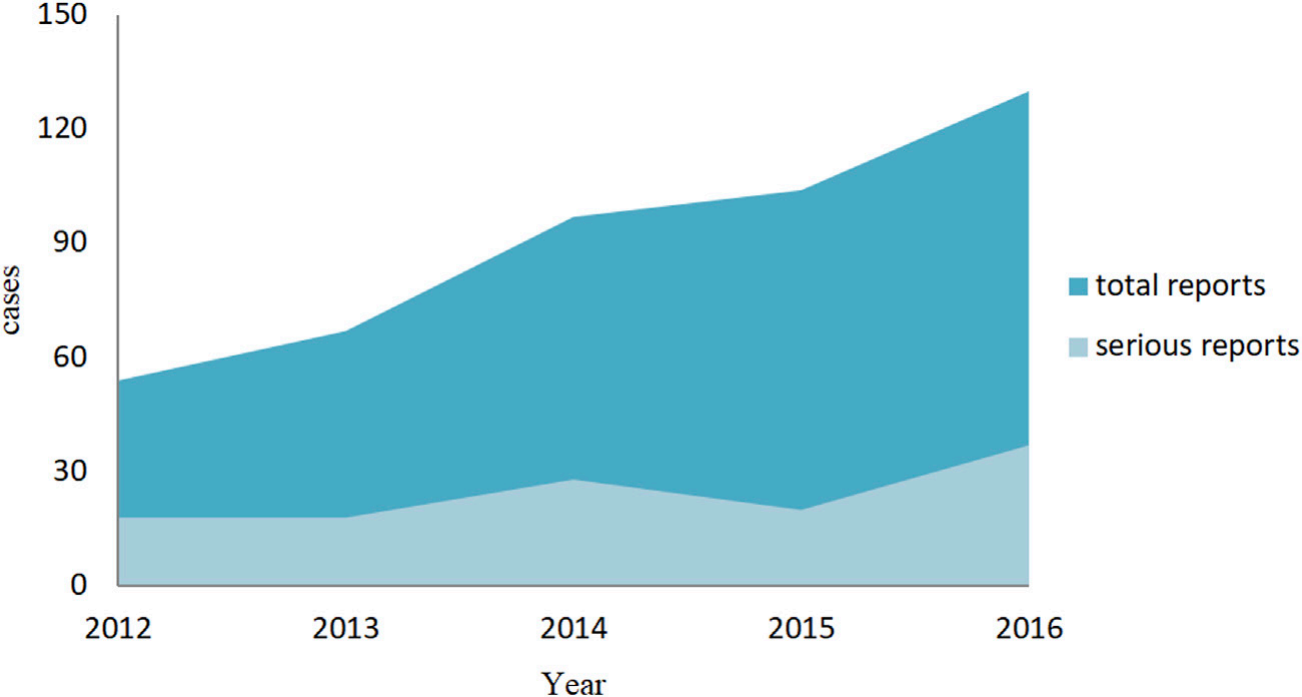
Annual distribution of aripiprazole-induced liver injury ADR reports.

We finalized a total of 452 valid reports, and among them, 121 cases were of a serious reactions (including 2 follow-up reports), and 331 were general reports. In addition to abnormal liver biochemical indicators, abnormal liver function, liver damage, and so on, while some patients are also accompanied by nausea, vomiting, and weight gain. When observing the overall trend of the number of reports, we have found that the ADR reports related to aripiprazole-induced liver injury show an increasing trend year by year. However, although there is also an increase in severe reports, the growth trend is relatively moderate, as shown in Figure 2.

**FIGURE 3:**
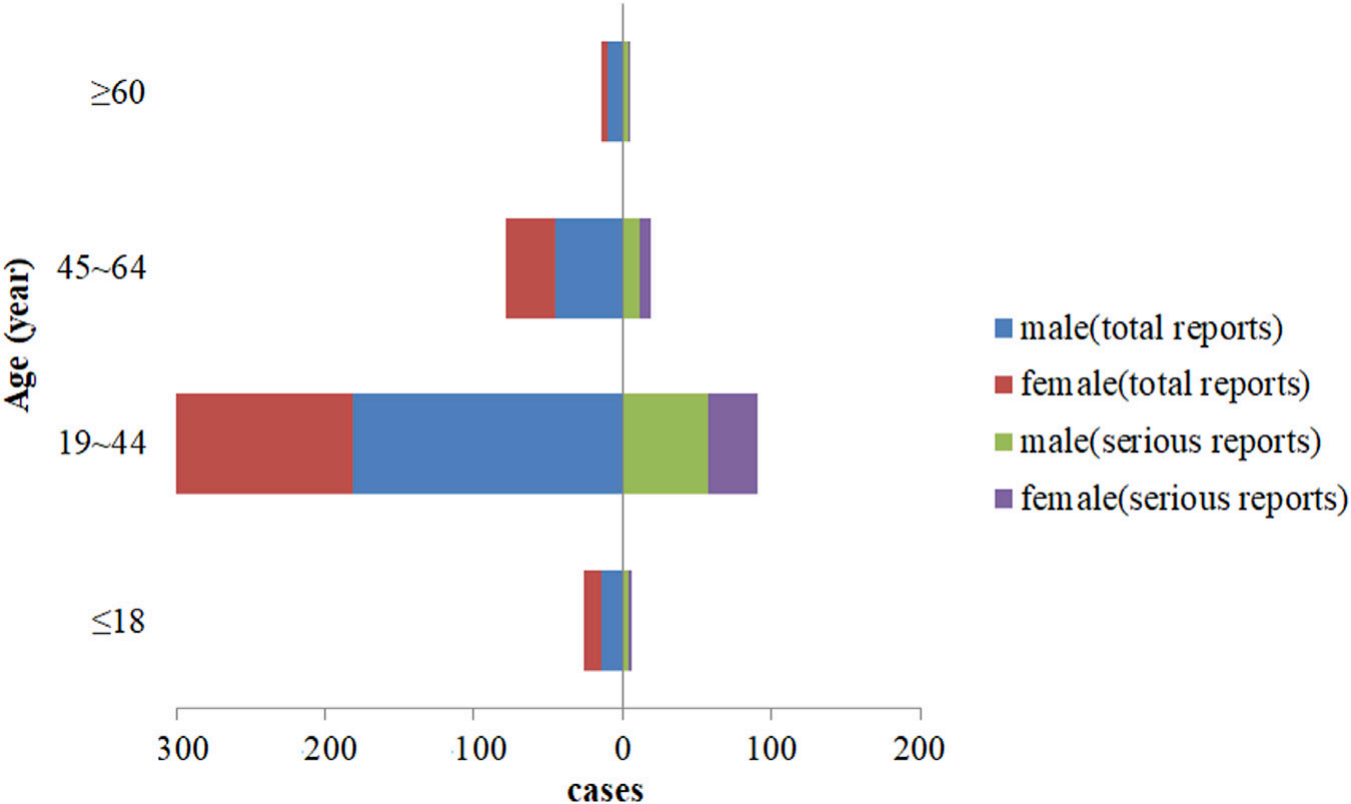
Comparison of patient gender and age distribution for different report types.

In the final analysis, there were 250 male patients and 202 female patients, with a male to female ratio of 1.24:1. The youngest was 11 years old, the oldest was 77 years old, and the average age was 100 (34.56 ± 12.81) years. Among the 121 serious reports, with a male-to-female ratio of 1.69:1, and the average age was (34.73 ± 13.16) years old, as shown in Figure 3.

### Underlying diseases and reasons for medication

In our analysis, we found that in addition to psychiatric illnesses, the various underlying diseases in patients included diabetes, hypertension, ankle fracture, coronary heart disease, lung abscess, moderate malnutrition, senile dementia, sleep disorders, and so on. The reasons for medication use were mainly treatment of schizophrenia, bipolar disorder, depression, hyperactivity attention-deficit syndrome, tic disorder, mania, and other psychiatric disorders. There was also one case each where the medication was prescribed for treating senile dementia, psychiatric, and behavioral disorders caused by alcohol use, and psychiatric and behavioral disorders caused by the use of multiple drugs and other psychoactive substances.

### Recovery and prognosis

After analysis, 356 of the 452 cases (78.76%) reported improvement or recovery after drug withdrawal or treatment. The recovery status of 96 cases was unknown, of which 95 reports belonged to patients who were given liver-protective treatment, dose reduction or (and) discontinuation of medication and other measures pending review, 1 case went to other hospitals for treatment due to aggravation of the original disease.

## Medication details

### Drug form and dosage

The adverse reaction reports mentioned three different formulations of aripiprazole: tablets, orally-disintegrating tablets, and capsules. The number of liver injury reports for each formulation were: 245 cases (54.20%) for tablets, 193 cases (42.70%) for orally-disintegrating tablets, and 14 cases (3.1%) for capsules. Based on the recorded the frequency and dosage of administration, it was found that the reports listed three dosing frequencies: once daily, twice daily, and thrice daily, with corresponding numbers of adverse reaction reports of 185 cases (40.93%), 258 cases (57.08%), and 9 cases (1.99%) respectively. The minimum and maximum daily doses reported were 1.25 mg and 30 mg respectively, with the most common dosage being 20 mg (161 cases, 35.62%), followed by 10 mg and 30 mg (95 cases, 21.02%) as shown in Table 1.

**TABLE 1:** Analysis of daily doses in adverse reaction reports of aripiprazole-induced liver injury.

Daily dose/D		Cases	Proportion (%)
**D** < **10 mg**	1.25 mg	1	0.22
	2.5 mg	4	0.88
	5 mg	17	3.76
**10 mg ≤ D ≤ 30 mg**	10 mg	95	21.02
	15 mg	36	7.96
	20 mg	161	35.62
	22.5 mg	1	0.22
	25 mg	19	4.20
	30 mg	95	21.02
**D > 30 mg**	40 mg	11	2.43
	45 mg	2	0.44
	50 mg	2	0.44
	60 mg	8	1.77
**Total**		452	100.00

### Medication combinations

There were 99 reports of combined medication, with some reports involving more than one medication. Overall, the most common types of medication combinations were other antipsychotics, sedatives-hypnotics, and antidepressants, followed by antidiabetic, antihypertensive, and antianxiety medications, as shown in Table 2. Additionally, after consulting the safety information in the instructions of the combined drugs, more than 70% of the adverse reactions were recorded as possibly causing liver injury. Paroxetine, perphenazine, duloxetine, and fluvoxamine are inhibitors of the liver enzymes CYP3A4 and CYP2D6.

**TABLE 2:** Medication combinations in 452 cases of aripiprazole liver ADR.

Drug category	Combined drug	Inhibitors of CYP3A4 or CYP2D6
Antipsychotic	Clozapine^a^, Olanzapine^a^, Quetiapine fumarate^a^, Perphenazine^a^, Risperidone^a^, Sulpiride^a^, Lithium carbonate, Amisulpride^a^, Haloperidol^a^, Penfluridol^a^	Perphenazine, Haloperidol
Sedative-hypnotic	Lorazepam^a^, Alprazolam^a^, Oxazepam, Estazolam^a^, Clonazepam^a^, Zopiclone^a^	
Antidepressive	Sertraline^a^, Escitalopram^a^, Fluvoxamine^a^, Paroxetine^a^	Paroxetine
Antidiabetic	Acarbose^a^, Glipizide^a^, Metformin hydrochloride	
Antiepileptic	Magnesium valproate^a^, Sodium valproate^a^	
Antianxiety	Buspirone^a^, Duloxetine^a^	Duloxetine
Antihypertensive	Propranolol^a^	
Narcotic	Propofol^a^	
Gastrointestinal	Dompendone/Domperidone	
Antiparkinsonian	Benzhexol	
Chinese patent drug	Jiuweigantai, Qiyeanshen, Tianmaxingnao, Xingnaojing	

^a^ Known drugs with liver injury (Instructions information or literature reported).

### Time of occurrence of adverse reactions

**FIGURE 4:**
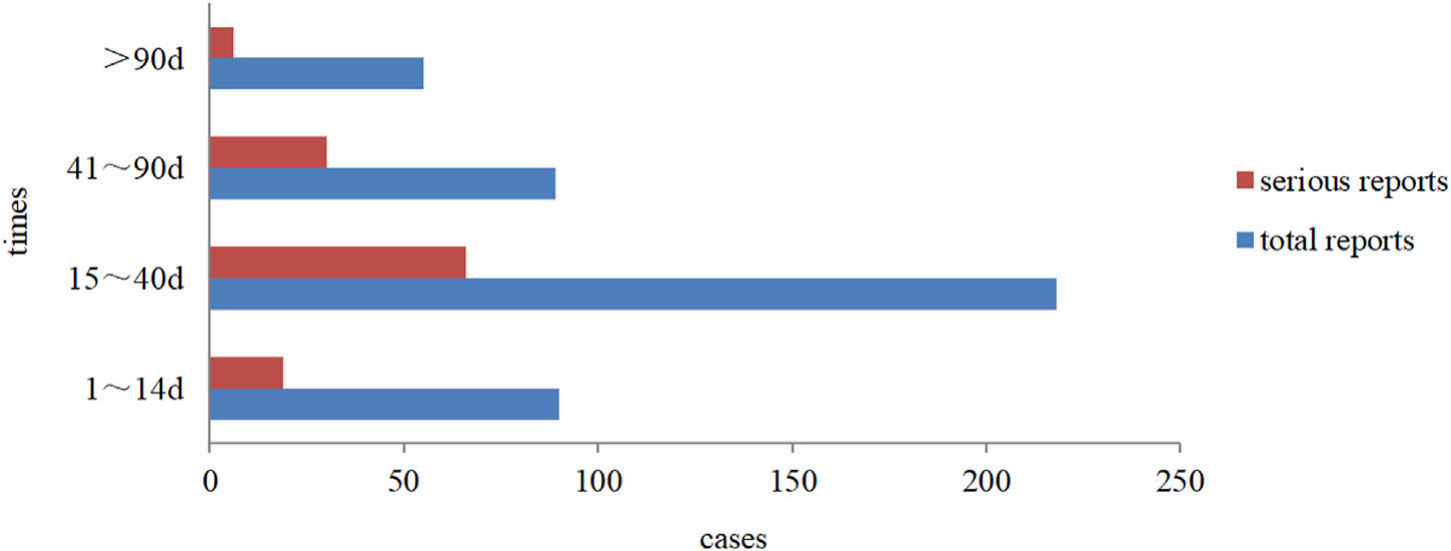
Temporal distribution of aripiprazole-induced liver injury.

Overall, drug-induced liver injury (DILI) with aripiprazole mainly occurred within 15–40 days after continuous use (about 50% of the cases). Severe reactions occurred within 3 months of medication, among which reactions between 15 and 40 days accounted for nearly 55%, and the distribution trend was basically consistent with the overall trend, as detailed in Figure 4. In addition, from the perspective of the time of occurrence of liver injury in different forms of aripiprazole, aripiprazole orally disintegrating tablets and aripiprazole tablets of liver injury had similar latency, also mainly within 15–40 days. Among the 14 cases of aripiprazole capsules, the onset time was within 1–14 days and 15–40 days in 6 cases each.

## Discussion

### Gender and age distribution

To our study, we found that there were slightly more male patients than female patients with aripiprazole-induced DILI. As per age distribution, majority of cases were young adults (19–44 years old) (accounting for about 74%), and the trend of gender and age distribution in reports of serious reactions was the same as the overall trend. According to an epidemiological survey in China, the average age of onset of schizophrenia was (27 ± 9) years old, the average age at first diagnosis and treatment was (32 ± 11) years old, and there was no significant difference in gender distribution (Huo et al., 2021). The distribution of gender and age in the population with aripiprazole-induced DILI in this study may be related to the morbidity characteristics of the disease for which aripiprazole was administered as treatment. Therefore, given the epidemiological characteristics of the population receiving aripiprazole treatment, the correlation between aripiprazole-induced DILI-related adverse reactions and patient gender and age requires further observation and research.

### Analysis of off-label drug use

When we analyzed the reasons for medication, there were individual reports of off-label use for conditions, such as mental and behavioral disorders caused by senile dementia, depression, attention-deficit hyperactivity syndrome, and tic disorder. Among them, the use of aripiprazole among senile dementia-related psychiatric patients could increase the risk of death, and there is a clear warning about this in the instructions, while some researchers have summarizes the findings of a comparative effectiveness review by the Agency for Healthcare Research and Quality, such as depression, dementia, anxiety, attention-deficit hyperactivity disorder, MDD monotherapy, substance abuse and Tourette’s syndrome and so on. Most of those uses lack of controlled trials, which highlights the need for new studies assessing the evidence for the use of aripiprazole in different clinical conditions (Maglione et al., 2011).

In terms of dosage, instructions for the usage of aripiprazole suggests an initial dose of 10 mg once daily for adults, which after 2 weeks of medication, can be gradually increased to a maximum of 30 mg/day according to the individual’s response and tolerance, and thereafter, this dose can be maintained. However, this study analysis indicates that the drug is used beyond the maximum recommended dose in clinical practice (23 cases, 5.09%), which may increase the risk of liver injury. Also, aripiprazole metabolism occurs in the liver by N-dealkylation, hydroxylation, dehydrogenation as well as the cytochrome P450 (CYP) 3A4 and 2D6 enzyme pathways, the lack of good drug tolerance in individuals can increase the risk of liver injury from aripiprazole (Chinese medical association et al., 2020; Prommer, 2017). In summary, these findings suggest that failure to use aripiprazole in accordance with the instructions is a non-negligible risk factor for adverse reactions to liver injury.

### Time of onset of DILI and recovery from ADR

Through the analysis of reports of various types and the time of occurrence of DILI caused by different dosage forms of aripiprazole, we found that the time of occurrence in all cases was from 15 to 90 days (about 67.92% of the time) after continuous usage. This may be related to the fact that most of the clinical increases in the dose of this drug were done incrementally after 2 weeks of administration, and it may also be related to the length of the course of drug administration (Zhou et al., 2020). In terms of recovery, 78.76% of patients improved or recovered after discontinuation of aripiprazole or suitable treatment, and this finding was consistent with the ease of recovery from the effects of aripiprazole on liver function that is reported in literature (Wang et al., 2014). It is thus clear that the period from 2 weeks to 3 months after starting aripiprazole is the most frequent period of liver injury. This also suggests that in clinical practice, particular attention should be given to monitoring aripiprazole-related liver injury within 2 weeks–3 months after medication, and most of them with adverse reactions will improve or recover with timely withdrawal or liver protection treatment after the ADR onset.

### Analysis of medication combinations

One study suggests that patients may experience severe DILI when taking aripiprazole with other liver injury medications (Pirc et al., 2020). In the adverse reaction reports we collected that involved combined drugs, 70% of the combined drug instructions listed that may cause liver injury. This indicates that our study results are consistent with reports from other studies, and its strongly suggests that the combination use of drugs known to cause liver injury is a risk factor for aripiprazole-induced liver injury. Therefore, monitoring liver functions is important in the cases when aripiprazole is co-prescribed or used with drugs with potential hepatotoxic effects.

In addition, the aripiprazole specifications indicate that is primarily metabolized by CYP3A4 and CYP2D6, where CYP3A4 or CYP2D6 inhibitors may inhibit the elimination of aripiprazole (Goodnick and Jerry, 2002; FDA, 2023), leading to an increase in blood drug concentration, enhanced pharmacological activity, and ultimately an increased risk of adverse reactions. However, most antipsychotic drugs are metabolized in the liver by cytochrome P450 enzymes such as CYP2D6 and CYP3A4 (Lv and Yi, 2018). These enzyme subtypes can cause different drug interactions and genetic effects of drugs, leading to pharmacokinetic interactions that can exacerbate or even induce drug-induced hepatotoxicity and alter the risk of adverse events (Urichuk et al., 2008; Woroń et al., 2018). In this study, combined drugs such as paroxetine, perphenazine, duloxetine, and haloperidol were inhibitors of CYP3A4 or CYP2D6. The reason for liver injury in these patients may be that the interaction between the drugs reduces the metabolic rate of aripiprazole and increases its exposure time in the liver, thereby inducing liver damage.

Therefore, it can be preliminarily speculated that the combination use of drugs known to cause liver injury and inhibitors of CYP3A4 or CYP2D6 is a potential risk factor for inducing liver injury with aripiprazole. When these factors exist, close attention should be paid to the clinical manifestations of patients after drug administration, monitoring of liver function, and actively preventing the occurrence of the risk of liver injury.

### Analysis of other risk factors

Although, in general, atypical antipsychotics rarely cause severe hepatotoxicity (Castanheira et al., 2019), it has been reported that patients with a history of alcohol consumption and drug (addictive substance) abuse who are put on aripiprazole may develop severe DILI (Pirc et al., 2020). In this study, there was one case each of mental and behavioral disorders caused by alcohol as well as other psychoactive substances. In the description of the course of adverse reactions, there were 5 reports that mentioned history of alcohol consumption, while we did not find any records related to history of drug abuse. Three of these 6 reactions were serious, with the shortest time for onset of the adverse reaction being 20 days and the longest being 63 days after drug administration. This finding is consistent with the trend of reduced/decreased hepatocyte division observed after 2 weeks of medication that is reported in literature (Pirc et al., 2020). These results suggest that for patients with a history of chronic alcohol consumption and drug (addictive drug) abuse, close monitoring of liver function is required during the use of aripiprazole to reduce the occurrence of severe drug-induced liver injury (DILI).

## Conclusion

To our knowledge, this is the first time that aripiprazole-induced liver injury has been analyzed using spontaneous reporting of ADR Big Data. Our study findings indicate that the number of adverse drug reaction (ADR) reports related to aripiprazole-induced liver injury has been increasing over the years, with slightly more male patients than female. ADRs occurring mainly within 15–90 days of medication use and the age distribution ranging from 19 to 44 years. The risk of aripiprazole-induced liver injury may be increased when taken in combination with known hepatotoxic drugs, CYP3A4 or CYP2D6 inhibitors, and other factors.

However, this study has some limitations. Due to the inherent issues of spontaneous reporting data, it is inevitable that there may be some omissions or inaccurate data, at the same time, as a retrospective analysis serving as a warning signal, we insisted on the inclusion of the original data as far as possible, which may lead to some false positive results. Therefore, the aripiprazole-induced liver injury risk should be further carried out by a prospective study to comprehensively analyze and assess, in order to identify and summarize the risk factors to promote its safe use in clinical practice.

## Data Availability

The raw data supporting the conclusion of this article will be made available by the authors, without undue reservation.
